# Drop-Set Training Elicits Differential Increases in Non-Uniform Hypertrophy of the Quadriceps in Leg Extension Exercise

**DOI:** 10.3390/sports9090119

**Published:** 2021-08-29

**Authors:** Dorian Varović, Kristian Žganjer, Saša Vuk, Brad J. Schoenfeld

**Affiliations:** 1 Faculty of Kinesiology, University of Zagreb, 10000 Zagreb, Croatia; dorian.varovic@student.kif.unizg.hr (D.V.); kristian.zganjer@student.kif.hr (K.Ž.); sasa.vuk@kif.unizg.hr (S.V.); 2 Department of Health Sciences, Lehman College, Bronx, NY 10468, USA

**Keywords:** muscle growth, training volume, resistance training, training methods, muscle adaptation

## Abstract

The study aimed to compare the effects of drop set resistance training (RT) versus traditional RT on markers of maximal muscle strength and regional hypertrophy of the quadriceps femoris. Sixteen recreationally active young men had one leg randomly assigned to the drop-set method (DS) and the other to training in a traditional manner (TRAD). Participants performed unilateral seated leg extensions using a periodized approach for eight weeks. Rectus femoris (RF) and vastus lateralis (VL) muscle thickness (MT), estimated one repetition maximum (RM) in the unilateral knee extension, and peak and average isokinetic knee extension torque at 60°/s angular velocity were measured pre- and post-study. Both conditions increased muscle thickness of the RF and VL from pre- to post-intervention. DS showed statistically greater increases in the RF at 30% and 50% of muscle length, whereas no MT differences were detected at 70% muscle length nor at any aspect of the VL. Both DS and TRAD increased estimated one RM from pre- to post-study (+34.6% versus +32.0%, respectively) with no between-condition differences noted. Both conditions showed similar increases in peak torque (DS: +21.7%; TRAD: +22.5%) and average torque (DS: +23.6%; TRAD: +22.5%) from pre- to post-study. Our findings indicate a potential benefit of the drop-set method for inducing non-uniform hypertrophic gains in the RF muscle pursuant to leg extension training. The strategy did not promote an advantage in improving hypertrophy of the VL, nor in strength-related measures, compared to traditional training.

## 1. Introduction

Resistance training (RT) is well-established as a primary interventional strategy for increasing muscle strength and mass across populations. Current theory indicates that RT-induced muscular adaptations can be optimized by manipulating exercise variables such as volume, load, and frequency, among others [1]. In an effort to further enhance adaptations, fitness enthusiasts often seek to employ a variety of advanced training strategies. One of the most popular of these strategies is the drop set method (a.k.a. breakdown sets) [2]. The drop set method involves performing a set to momentary muscular failure (MMF), then immediately reducing the load (generally by 20% to 25%) and performing as many additional repetitions as possible [3]. If desired, double or triple drops can be employed to heighten stimulation of working muscle fibers and thus perhaps enhance muscular adaptations [3]. 

The drop set method is largely based on the premise that muscles are not fully fatigued when sets are carried out to MMF, as they are still capable of producing force at lower loads [3]. Thus, performing additional repetitions at a decreased magnitude of load immediately after reaching muscle failure in a set may elicit heightened fatigue of muscle fibers, potentially leading to a superior anabolic response [4]. In addition, the combination of a high number of repetitions performed with minimal rest periods induces high levels of metabolic stress [2], which has been theorized as a potential stimulus for hypertrophic gains [5].

Longitudinal research is currently limited as to the effects of drop set training on muscular adaptations of the lower limb. Seminal work by Goto et al. [6] provided preliminary support for the strategy, showing significantly greater increases in one repetition maximum (RM) leg press and maximal isokinetic knee extension torque for a group performing a single drop set to MMF using 50% one RM compared to a traditional set configuration. Moreover, the drop set group realized a ~2% increase in thigh muscle cross sectional area whereas the traditional group displayed a slight loss in muscle size (~0.5%), although differences in this outcome did not rise to statistical significance (*p* < 0.08). While these results are intriguing, several issues must be taken into account when attempting to draw inferences from the data. For one, the sample was small and likely underpowered (17 total subjects in a parallel group design), raising the possibility of a Type II error. In addition, the drop set group performed more sets than the traditional group, which may have confounded hypertrophic adaptations [7]. Importantly, the drop set group took a 30-s rest period after the fifth set before initiating the drop set. Given that the drop set method customarily is performed with as little rest as possible between drops, the relevance to breakdown training is questionable.

More recently, Angleri et al. [8] randomized the lower limbs of resistance-trained men so that one leg performed a traditional training routine (three to five sets of six to twelve repetitions with a two-min inter-set rest interval) while the other leg performed the same protocol with up to two drop sets at a 20% reduction in load. After 12 weeks, participants achieved significant increases in measures of muscle strength and hypertrophy with no significant differences observed between conditions. It should be noted that hypertrophy was only assessed in the vastus lateralis (VL) at a single point along the length of the muscle (corresponding to 50% femur length). 

Research indicates that exercise-induced quadriceps femoris hypertrophy can manifest in a non-uniform fashion, with different magnitudes of growth observed in the proximal, middle, and distal regions of the musculature [9,10,11]. Thus, it is not clear whether differences may have existed in other regions of the VL, or in other muscles of the quadriceps. Given that drop-set training involves performing additional repetitions after muscle failure, it is conceivable that the strategy could stimulate aspects of muscles containing a higher proportion of fibers with predominantly oxidative characteristics, and consequently, from a theoretical point of view, this may contribute to greater hypertrophy in these sites. The purpose of the present study was to compare the effects of lower-limb drop set training versus traditional training on muscle strength and regional hypertrophy of the quadriceps in a sample of physically active young men. We hypothesized that the drop-set method would promote greater increases in strength and size of the quadriceps femoris compared to traditional training.

## 2. Materials and Methods

### Participants

Thirty-five young men were recruited from a population of healthy, physically active kinesiology students for possible participation in this study. Prospective participants were considered eligible if they had less than 1 year of RT experience and no lower extremity injuries. The sample size was justified by a priori power analysis using G*Power software (Germany, Düsseldorf, version 3.1.9.7) based on an effect size (ES) of 0.40 for vastus lateralis (VL) MT, an alpha level of 0.05, and a power (1−β) of 0.80, consistent with findings by Lasevicius et al. [12]. Analysis showed the required sample size to achieve sufficient statistical power was 15 participants. 

After attending an initial informational and explanatory meeting, five participants did not meet the required inclusion criteria, and six others decided not to enroll in the study for personal reasons. Thus, a total of 24 participants (age: 19.21 ± 1.10 years; height: 183.13 ± 5.55 cm; body weight: 78.50 ± 7.11 kg) agreed to participate in the training intervention. All participants were informed of potential risks and benefits of the study and signed an informed consent prior to participation. This study was approved by the Committee for Scientific Research and Ethics of the local university and conducted in accordance with the Declaration of Helsinki. 

**Figure 1:**
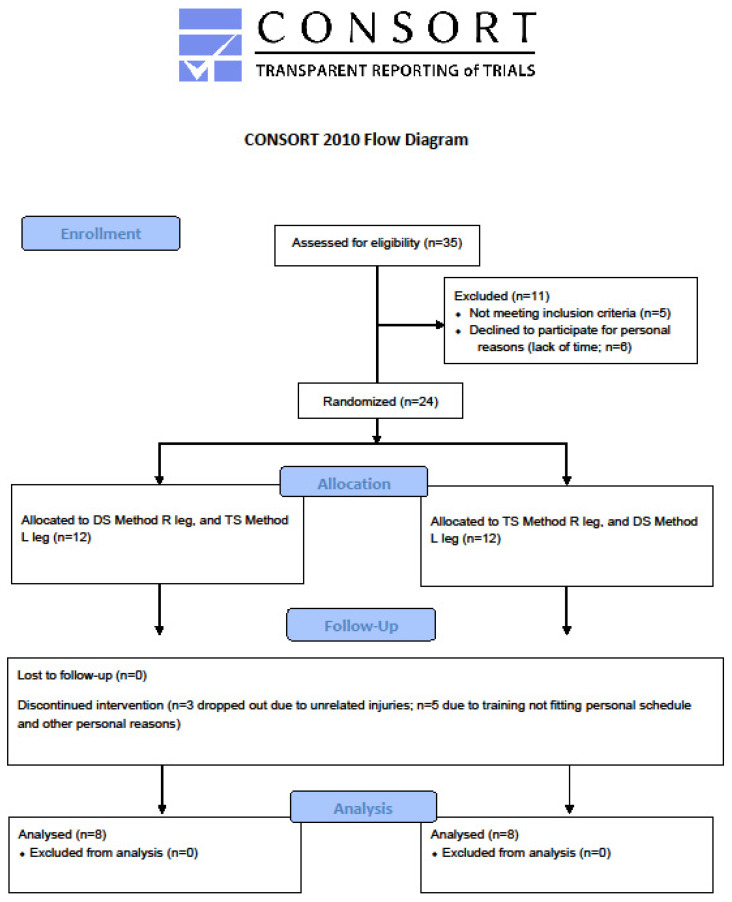
CONSORT flow diagram showing progress of participants through all phases of the study.

Prior to participation, the participants legs were randomly assigned to one of two conditions: drop set (DS) or traditional (TRAD) training. Participants had to attend at least 90% of the training sessions (absence in a total of two non-consecutive training sessions were allowed) for their data to be included in the final analysis. The participants were instructed to refrain from any intensive physical activities (outside the study program) and to maintain their current eating habits for the duration of the study. The CONSORT flow diagram is presented in Figure 1. 

## 3. Procedures

### 3.1. Muscle Thickness

Muscle thickness (MT) measurements of the m. vastus lateralis (VL) and m. rectus femoris (RF) were carried out before and after the RT intervention via B-mode ultrasonography (Siemens, SONOLINE G-40). Participants were instructed to refrain from any intensive exercise for at least 48 h before the testing session to prevent the possible confounding of results from muscle swelling [13]. Ultrasound imaging was conducted by an experienced technician using a linear 10–5 MHz probe. Testing was carried out with participants in a supine position with knees slightly flexed. The length of the RF was measured as the distance between the anterior superior iliac spine (ASIS) and the upper aspect of the patella. The length of the VL was measured as the distance between the greater trochanter and the upper aspect of the patella. Measurements were taken at three different sites corresponding to 30% (proximal), 50% (mid) and 70% (distal) along the muscle length. Each site was marked with semi-permanent ink to help ensure consistency of the measures. The technician applied water soluble transmission gel to each measurement area and placed the probe perpendicular to the muscle tissue, taking caution to avoid depressing the skin. When the quality of the image was deemed satisfactory, the image was saved to the ultrasound unit’s internal hard drive. Two separate images were obtained for each measurement site. 

Analysis of ultrasound images was carried out using ImageJ software (version 1.53c; National Institutes of Health, Bethesda, MD, USA). MT was determined as the distance between the superficial aponeurosis and the deep aponeurosis [14]. The average of the two images for each site was calculated as the final value. The ultrasound technician was blinded to the study conditions both during imaging and analysis. The intraclass correlation coefficients for ultrasound measurements across sites ranged from 0.92–0.99. 

### 3.2. Muscle Strength—Isokinetic Dynamometry

Maximal concentric muscle strength was assessed via isokinetic dynamometry (System 4, Biodex Corporation, Shirley, New York, NY, USA). The selected test was the unilateral knee extension at 60°/s of angular velocity for three repetitions. Prior to testing, each participant performed a standardized dynamic five-min warm-up consisting of various track-and-field drills (skips, high steps, lateral crossovers), 12–15 bodyweight squats and 12 lunges with each leg. Thereafter, participants were placed in a seated position in the dynamometer with their trunk and distal thigh stabilized by belts and their arms crossed across the chest to eliminate any extraneous body movements. The machine’s lever arm was individually adjusted to accommodate the length of each participant’s legs. Specifically, the mechanical axis of the dynamometer was aligned with the lateral epicondyle of the femur, and the ankle pad was set proximal to the lateral malleolus. Calibration of the dynamometer was conducted before each testing session, after which range of motion ranged from 90° (flexion) to 170° (extension). Additionally, gravity-corrected torque was performed for each leg prior to the testing procedure along with leg’s weight being measured at an extension of 150°. Participants were instructed to perform all repetitions with maximum effort; the research staff provided verbal encouragement throughout each trial. All participants performed two submaximal repetitions followed by three repetitions with maximal effort. The peak torque at knee extension was used as the final value for this outcome. Peak torque was reported as the highest torque output that subjects achieved while performing the repetitions (out of three). Average peak torque was reported as the average value of the three repetitions.

### 3.3. Muscle Strength—Estimated One RM 

To avoid the potential confounding effects of fatigue from previous isokinetic testing, we carried out estimated one RM leg extension testing on a separate day. Each participant performed a standardized dynamic three-min warm up prior to testing consisting of various track-and-field drills (skips, high steps, lateral crossovers), dynamic stretching of the lower extremities, and 12–15 bodyweight squats. Estimation of one RM followed the Brzycki equation using a five RM, which has shown to be a valid option for assessing maximal strength [15] as per the following equation:

Weight lifted/(1.0278 − 0.0278X)

where X is the number of repetitions performed

We chose to estimate one RM via submaximal loading as opposed to maximal loading due to the increased risk of injury with very heavy loads in a single joint exercise [16], which is of particular issue in novice trainees. After assessing estimated one RM for each participant in the pre-study testing session, five RM (89% of 1 RM) and fifteen RM (67% of one RM) were also calculated to determine starting loads for the DS and TRAD conditions, respectively.

## 4. Resistance Training Protocol

During the eight-week RT intervention, participants trained under direct supervision of personal trainers. The routine consisted of the leg extension exercise, with the number of sets gradually increasing each week. Participants performed a standardized five-min warm-up prior to each training session that consisted of various track-and-field drills (skips, high steps, lateral crossovers), 12–15 bodyweight squats and two sets on the leg extension machine for 8–10 repetitions with 50% of their estimated one RM. The program followed a linear periodization model that readjusted training loads based on each participant’s progression rate. The starting leg was alternated from one session to the next in counterbalanced fashion so that neither condition obtained a performance advantage over time. Once participants completed all the repetitions on one leg, they immediately performed the alternate condition on the contralateral leg, then rested for 120 s before performing the ensuing set. Participants were coached to perform concentric and eccentric actions with a cadence of 1:2 s. 

The DS protocol involved performing sets with a ~five RM load carried out to MMF. Immediately thereafter, the load was reduced by 20% and participants continued to perform additional repetitions until reaching MMF, at which point the load was reduced by 10–15% and the set was concluded once the participant reached MMF at that load. To account for strength progression over time, we set a target repetition range of three to seven repetitions in which the participant would have to reach MMF with a given load. Loads were continually adjusted to maintain this target repetition range. Alternatively, the TRAD protocol involved performing sets with a ~15 RM load with repetitions carried out consecutively until the participant reached MMF. To account for strength progression over time, we targeted a range of 13 to 17 repetitions with loads continually adjusted from set to set so as to maintain this repetition range. 

The total training volume, determined as the number of sets per session, was gradually increased from week to week with participants performing the same number of sets for both conditions. In the first week of the study, participants performed only one training session consisting of three sets in total; in the second week they performed two training sessions consisting of four sets per session. This gradual increase in volume facilitated adaption to the training stimulus so as to minimize the potential for delayed onset muscle soreness (DOMS) and establish a repeated bout effect whereby DOMS would not interfere with training performance [17]. Thereafter, participants performed three weekly sessions for the duration of the intervention. The total number of sets peaked in Week 7, with participants performing a total of 15 working sets throughout the week. Volume was tapered in the eighth week to promote recovery and restoration (see Table 1).

## 5. Statistical Analysis

Statistical analyses were carried out using SPSS Statistics software for Windows (version 27.0; IBM Corp.) and Excel 365 (Microsoft Corp.), with data expressed as mean and standard deviation (SD) values. We used a two-way, repeated-measures ANOVA to test for the time (pre and post intervention) x condition (experimental and control) interaction. If no statistically significant interaction was detected, a Bonferroni correction was used to determine the presence of main effects. A paired samples T-test was employed to determine whether differences in total training volume existed between the two conditions. Cohen’s d effect size (ES) was calculated as the mean pre-post change divided by the pooled SD. ESs of 0.00 to 0.19, 0.20 to 0.49, 0.50 to 0.79, and >0.80 were considered to represent trivial, small, moderate and large effects, respectively [18]. In addition, percent changes were calculated as the mean pre-post change divided by the pre-study mean multiplied by 100. Cumming estimation plots were created for MT data using computer-based software [19]. The statistical significance level was set a priori at *p* < 0.05.

## 6. Results

### 6.1. Participants

From the initial 24 participants who began the study, 8 dropped out prior to completion: 5 dropped out for personal reasons (could not sustain training volume, lost interest because lack of time, wanted to focus on studies), while 3 dropped out due to injuries sustained outside the intervention. Thus, a total of 16 participants were ultimately included in the final analysis.

### 6.2. Muscle Strength

Mean ± SD, effect size (ES), and percentage of change in tests of maximum strength from pre- to post-intervention are shown in Table 2.

For the one RM test, there was a significant main effect for time (*p* < 0.001) but no main effect for group (*p* = 0.483). We did not observe a significant group x time interaction (*p* = 0.378). For peak torque we observed significant main effects for both factors, group (*p* = 0.016) and time (*p* < 0.001) without a significant group x time interaction (*p* = 0.988). For average peak torque there was a significant main effect favoring time (*p* < 0.001), but not a significant main effect for group (*p* = 0.058) nor a group x time interaction (*p* = 0.783).

### 6.3. Muscle Thickness

**Figure 2:**
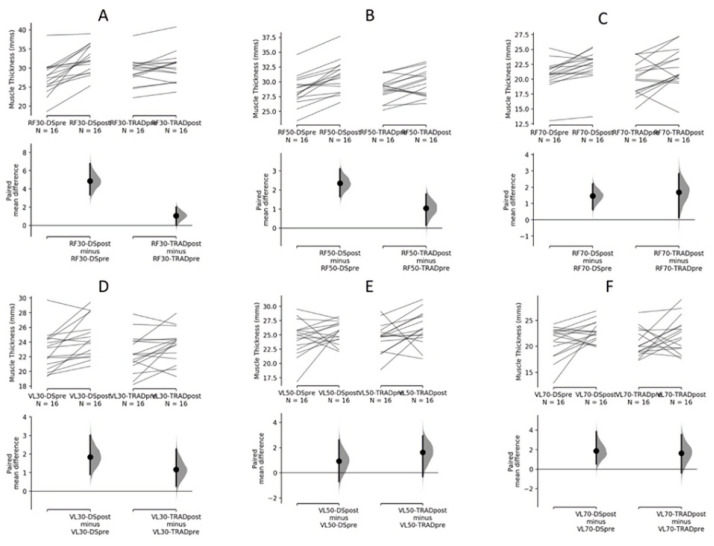
Estimation plot of the paired mean difference for hypertrophic responses for RF proximal (30%-**A**), RF middle (50%-**B**), RF distal (70%-**C**), VL proximal (30%-**D**), VL middle (50%-**E**), VL distal (70%-**F**) portions of the thigh at within-subjects (**top**) and between-groups (**bottom**) conditions. The raw data are plotted on the upper axes; each paired set of observations is connected by a line. On the lower axes, each paired mean difference is plotted as a bootstrap sampling distribution. Mean differences are depicted as dots; 95% confidence intervals are indicated by the ends of the vertical error bars. DS = drop-set; TRAD = traditional resistance training.

Mean ± SD, effect size (ES), and percent change in MT of the RF and VL from pre- to post-intervention are shown in Table 2. A Cumming estimation plot of individual and mean data are shown in Figure 2.

For the RF at 30% muscle length, we observed a significant main effect for time (*p* < 0.001) as well as a group × time interaction favoring DS (*p* = 0.001); we did not observe a main effect for group (*p* = 0.80). For the RF at 50% muscle length, we observed a significant main effect for time (*p* < 0.001) as well as a group x time interaction favoring DS (*p* = 0.034); we did not observe a significant main effect for group (*p* = 0.25). For the RF at 70% muscle length, we observed a significant main effect for time (*p* = 0.006). We did not observe a significant main effect for group (*p* = 0.39) nor a group × time interaction (*p* = 0.70).

For the VL at 30% muscle length, we observed a significant main effect for time (*p* < 0.001); we did not observe a significant main effect for group (*p* = 0.14) nor a group × time interaction (*p* = 0.439). For the VL at 50% muscle length, we did not observe any significant main effects nor any group × time interaction (ES = 0.24). For the VL at 70% muscle length, we did not observe any significant main effects nor any group × time interaction (*p* = 0.874).

### 6.4. Total Training Volume

Paired samples *t*-test revealed no statistically significant differences in total training volume (calculated as sets × reps) between the conditions (*p* = 0.92). However, volume load (calculated as reps × sets × load) showed statistically significant differences between conditions favoring DS (*p* > 0.001).

## 7. Discussion

This study aimed to compare the effects of drop-set training with traditional training on measures of muscle strength and hypertrophy. A novel finding was that DS elicited superior hypertrophy of the RF muscle in a non-uniform manner. Alternatively, hypertrophy of the VL was similar between conditions. In addition, DS did not appear to enhance strength-related adaptations compared to TRAD. We discuss the implications of our findings below.

### 7.1. Muscle Thickness

Assessment of changes in MT showed that DS promoted greater growth in two of the three measurement sites of the RF compared to TRAD; alternatively, changes in MT of the VL were statistically similar between conditions. Differences in RF hypertrophy were most pronounced in the proximal portion (30% of muscle length), where DS elicited a 17.7% increase versus a 3.7% increase for TRAD. The effect size difference equated to 0.87, indicating a large magnitude of effect. DS also produced greater increases at the midpoint of the RF (50% of muscle length) compared to TRAD, although the relative magnitude of differences was smaller (8.3% versus 3.6%, respectively) with a moderate effect size difference (0.58). These results suggest that drop set training may help to enhance regional hypertrophy of the rectus femoris. No hypertrophic differences between conditions were observed for the distal portion of the RF.

Our findings expand on those of Angleri et al. [8], who found similar increases in cross sectional area of the VL when training in a traditional fashion versus employing drop sets. Importantly, Angleri et al. [8] only assessed hypertrophy in the VL at a single-site (midpoint of the muscle) whereas we evaluated both the VL and RF along multiple aspects of the muscle length. Consistent with the results of Angleri et al. [8], we found no differences in muscle thickness at the midpoint of the VL between conditions; in addition, we did not observe any differences in the proximal and distal regions of the muscle as well. Alternatively, we demonstrated that greater hypertrophic increases occurred in the proximal- and mid-regions of the RF with the use of drop set training; Angleri et al. [8] did not assess changes in this muscle, precluding comparison between studies.

Although we did not attempt to determine potential mechanistic explanations for our findings, we can hypothesize that the observed differences in MT between the various segments of the RF and VL muscles may be related to the use of a single-joint knee extension exercise. Emerging research indicates that single-joint knee extension RT preferentially activates the RF [20,21], which in turn may induce greater growth of this muscle with consistent training [22]. This may be due the fact that the RF is placed under loaded stretch during leg extension exercise, which has been shown to enhance hypertrophic adaptations [23]. Our results further this line of research, providing evidence that drop set training may in fact enhance increases in RF muscle mass without having such an effect on the VL. Although speculative, it is possible that the drop set method heightened mechanical stress to the RF, given that the muscle was trained in a lengthened position with a higher volume load. This hypothesis warrants further study.

Notably, we provide additional evidence that RT-induced hypertrophy of the quadriceps femoris can occur in a non-uniform fashion. Our findings indicate that changes in muscle size varied considerably along the length of the muscles studied, as demonstrated in previous research [9,10,11]. Thus, to obtain a true understanding of muscular adaptations pursuant to longitudinal RT designs, researchers should endeavor to measure hypertrophy at proximal, middle, and distal sites when investigating hypertrophic changes of the quadriceps.

### 7.2. Muscle Strength

Both set configurations elicited similar increases in strength. Large changes were observed for DS and TRAD in the estimated one RM test (34.6% vs. 32.0%, respectively) as well as dynamometry-assessed isokinetic peak torque (21.7% vs. 22.5%, respectively) and average torque (23.6% vs. 22.5%, respectively). No statistical differences were observed between conditions, indicating that DS training does not confer an advantage for improving strength-related measures. Our findings are consistent with those of Angleri et al. [8], who reported significant increases in the one RM leg press and leg extension after twelve weeks of drop set vs traditional training, with no observed differences between conditions.

It is interesting to note that the initial loads for each set in our study were substantially heavier in DS compared to TRAD (~five RM vs. ~fifteen RM). Given research showing a strength-related benefit to the use of heavier loads [7], it therefore, might be expected that the DS group would have outperformed TRAD on tests of force capacity. However, this was not the case here. One possible explanation for the finding is that we employed a submaximal strength test to estimate one RM. Based on the principle of specificity, it can be speculated that transfer of strength would be greatest when training as close to maximum loading capacity as possible. Our test could be considered more of a “strength-endurance” assessment as opposed to a pure maximal strength test (i.e., true one RM). Similarly, the employed isokinetic tasks lacked specificity to the training protocols, which involved using a dynamic constant external resistance exercise. Evidence indicates that strength-related changes between loading ranges are mitigated when testing is carried out on a device dissimilar to that used during the exercise intervention [7], thus providing a viable rationale for the lack of observed difference between conditions.

### 7.3. Limitations

Our study had several limitations that should be considered when attempting to draw practical inferences. First, results are specific to the leg extension exercise and thus cannot necessarily be extrapolated to compound lower body movements or exercises/muscles for other areas of the body. Second, the results are specific to recreationally trained young men and cannot necessarily be generalized to women, adolescents, the elderly, or those with considerable RT experience. Third, the study had a relatively short duration of eight weeks; although this time-frame has consistently proven sufficient to elicit robust increases in muscle strength and hypertrophy, as was the case in our study, it is unclear whether results might have changed had training been carried out over longer time periods. Fourth, we estimated one RM using submaximal testing methods; although the employed formula has been validated for assessment of maximal dynamic strength [15], it is susceptible to error and thus may have influenced extrapolation of results on this outcome. Finally, although the within-subject design can be considered a study strength in that it reduces variability and hence increases statistical power, it remains possible that strength adaptations may have been confounded by a cross-education effect [24]. A number of studies have demonstrated that training one limb results in a strength increase of the contralateral limb, conceivably via modifying motor pathways that innervate the opposite limb [24]. However, evidence of such an effect occurring is limited to an untrained contralateral limb versus when both limbs perform an RT intervention. No study to date has shown that training both limbs with different methods results in a cross-education effect, raising skepticism that this phenomenon unduly influenced results. Moreover, the body of evidence does not seem to indicate that cross education appreciably affects measures of muscle hypertrophy in an untrained contralateral limb [25], and the possibility seemingly would be even less likely in the case where both limbs are trained. We recommend replication of the study with a parallel design to rule out potential confounding from cross-education.

## 8. Practical Applications

The present study indicates a potential benefit of the drop-set method for inducing non-uniform hypertrophic gains in the RF muscle pursuant to leg extension training. The strategy did not promote an advantage in improving hypertrophy of the VL, nor in strength-related measures compared to traditional training. From a practical standpoint, practitioners may consider implementing lower body drop set training using the leg extension when the goal is to achieve maximum quadriceps hypertrophy or specifically target the RF muscle. Future research should seek to determine how such training can best be implemented to optimize results.

## Figures and Tables

**Table 1:** Training volume (number of sets) progression throughout the study duration.

Time Period	Monday	Wednesday	Friday
Week 1	-	-	3
Week 2	4	4	-
Week 3	4	4	3
Week 4	4	4	4
Week 5	5	4	4
Week 6	5	5	4
Week 7	5	5	5
Week 8	3	2	2

**Table 2:** Mean ± SD, effect size (ES) and percentage change (%) from pre to post intervention in assessments of strength and muscle thickness.

Variable	Condition	Pre Mean ± SD	Post Mean ± SD	*p* (Group)	*p* (Time)	*p* (Time × Group)	ES (d)	Change (%)
**One RM (kg)**	DS	71.43 ± 6.86	96.18 ± 16.07	0.483	<0.001 †	0.378	2.00	34.6
	TRAD	71.62 ± 7.04	94.51 ± 13.12				2.17	32.0
**PTQ (Nm)**	DS	209.14 ± 37.41	254.49 ± 37.44	0.016	<0.001 †	0.988	1.21	21.7
	TRAD	200.84 ± 24.40	246.03 ± 41.72				1.32	22.5
**ATQ (Nm)**	DS	197.58 ± 40.12	244.15 ± 33.05	0.058	<0.001 †	0.783	0.20	23.6
	TRAD	193.26 ± 23.55	236.78 ± 43.73				1.24	22.5
**RF_30**	DS	27.75 ± 4.39	32.32 ± 3.71	0.80	<0.001 †	<0.001†	1.20	17.7
	TRAD	29.21 ± 3.65	30.28 ± 4.00				0.28	3.7
**RF_50**	DS	28.57 ± 2.55	30.93 ± 2.76	0.25	<0.001 †	<0.034†	0.89	8.3
	TRAD	28.78 ± 1.92	29.83 ± 2.25				0.50	3.6
**RF_70**	DS	20.78 ± 2.59	22.24 ± 2.67	0.39	<0.006 †	0.702	0.56	7.0
	TRAD	20.12 ± 2.79	21.81 ± 3.24				0.56	8.4
**VL_30**	DS	22.74 ± 2.66	24.59 ± 2.73	0.14	<0.001 †	0.439	0.69	8.1
	TRAD	22.44 ± 2.60	23.60 ± 2.28				0.47	5.2
**VL_50**	DS	24.51 ± 3.09	25.43 ± 1.88	0.56	0.051	0.580	0.36	3.8
	TRAD	24.52 ± 2.54	26.14 ± 2.87				0.60	6.6
**VL_70**	DS	20.92 ± 3.09	22.78 ± 1.99	0.091	0.687	0.053	0.72	8.9
	TRAD	20.63 ± 2.64	22.26 ± 3.36				0.54	7.9

DS = “drop-set” method; TRAD = traditional method; PTQ = knee extension peak torque; ATQ = knee extension average peak torque; RF = m. rectus femoris; VL = m. vastus lateralis; _30, _50, _70 = measuring site along the length of each muscle; † = significant at *p* < 0.05.

## Data Availability

Data can be obtained by contacting the lead author, Dorian Varovic, at: dorian.varovic@student.kif.unizg.hr.

## References

[1] Kraemer W.J., Ratamess N.A. (2004). Fundamentals of Resistance Training: Progression and Exercise Prescription. Med. Sci. Sports Exerc..

[2] Krzysztofik M., Wilk M., Wojdała G., Gołaś A. (2019). Maximizing Muscle Hypertrophy: A Systematic Review of Advanced Resistance Training Techniques and Methods. Int. J. Environ. Res. Public Health.

[3] Schoenfeld B.J., Grgic J. (2018). Can Drop Set Training Enhance Muscle Growth?. Strength Cond. J..

[4] Schoenfeld B. (2011). The Use of Specialized Training Techniques to Maximize Muscle Hypertrophy. Strength Cond. J..

[5] Schoenfeld B.J. (2013). Potential Mechanisms for a Role of Metabolic Stress in Hypertrophic Adaptations to Resistance Training. Sports Med..

[6] Goto K., Nagasawa M., Yanagisawa O., Kizuka T., Ishii N., Takamatsu K. (2004). Muscular adaptations to combinations of high- and low-intensity resistance exercises. J. Strength Cond Res..

[7] Schoenfeld B.J., Ogborn D., Krieger J.W. (2016). Dose-response relationship between weekly resistance training volume and increases in muscle mass: A systematic review and meta-analysis. J. Sports Sci..

[8] Angleri V., Ugrinowitsch C., Libardi C.A. (2017). Crescent pyramid and drop-set systems do not promote greater strength gains, muscle hypertrophy, and changes on muscle architecture compared with traditional resistance training in well-trained men. Graefe’s Arch. Clin. Exp. Ophthalmol..

[9] Blazevich A., Gill N.D., Zhou S. (2006). Intra- and intermuscular variation in human quadriceps femoris architecture assessed in vivo. J. Anat..

[10] Narici M.V., Roi G.S., Landoni L., Minetti A.E., Cerretelli P. (1989). Changes in force, cross-sectional area and neural activation during strength training and detraining of the human quadriceps. Graefe’s Arch. Clin. Exp. Ophthalmol..

[11] Narici M.V., Hoppeler H., Kayser B., Landoni L., Claassen H., Gavardi C., Conti M., Cerretelli P. (1996). Human quadriceps cross-sectional area, torque and neural activation during 6 months strength training. Acta Physiol. Scand..

[12] Lasevicius T., Schoenfeld B.J., Grgic J., Laurentino G., Tavares L.D., Tricoli V. (2019). Similar Muscular Adaptations in Resistance Training Performed Two Versus Three Days Per Week. J. Hum. Kinet..

[13] Ogasawara R., Thiebaud R.S., Loenneke J.P., Loftin M., Abe T. (2012). Time course for arm and chest muscle thickness changes following bench press training. Interv. Med. Appl. Sci..

[14] Reeves N.D., Maganaris C.N., Longo S., Narici M.V. (2009). Differential adaptations to eccentric versus conventional resistance training in older humans. Exp. Physiol..

[15] DiStasio T.J. (2014). Validation of the Brzycki and Epley Equations for the 1 Repetition Maximum Back Squat Test in Division I College Football Players. Ph.D. Thesis.

[16] Kanada Y., Sakurai H., Sugiura Y., Arai T., Koyama S., Tanabe S. (2017). Estimation of 1RM for knee extension based on the maximal isometric muscle strength and body composition. J. Phys. Ther. Sci..

[17] Cheung K., Hume P., Maxwell L. (2003). Delayed onset muscle soreness: Treatment strategies and performance factors. Sports Med..

[18] Cohen J. (1992). A power primer. Psychol. Bull..

[19] Ho J., Tumkaya T., Aryal S., Choi H., Claridge-Chang A. (2019). Moving beyond P values: Data analysis with estimation graphics. Nat. Methods.

[20] Andersen L.L., Magnusson S.P., Nielsen M., Haleem J., Poulsen K., Aagaard P. (2006). Neuromuscular Activation in Conventional Therapeutic Exercises and Heavy Resistance Exercises: Implications for Rehabilitation. Phys. Ther..

[21] Enocson A., Berg H., Vargas R., Jenner G., Tesch P. (2005). Signal intensity of MR-images of thigh muscles following acute open- and closed chain kinetic knee extensor exercise—Index of muscle use. Graefe’s Arch. Clin. Exp. Ophthalmol..

[22] Ema R., Wakahara T., Miyamoto N., Kanehisa H., Kawakami Y. (2013). Inhomogeneous architectural changes of the quadriceps femoris induced by resistance training. Graefe’s Arch. Clin. Exp. Ophthalmol..

[23] Oranchuk D.J., Storey A.G., Nelson A., Cronin J.B. (2018). Isometric training and long-term adaptations: Effects of muscle length, intensity, and intent: A systematic review. Scand. J. Med. Sci. Sports.

[24] Lee M., Carroll T.J. (2007). Cross education: Possible mechanisms for the contralateral effects of unilateral resistance training. Sports Med..

[25] Hendy A.M., Lamon S. (2017). The Cross-Education Phenomenon: Brain and Beyond. Front. Physiol..

